# Factors affecting upper airway control of NSAID‐exacerbated respiratory disease: A real‐world study of 167 patients

**DOI:** 10.1002/iid3.347

**Published:** 2021-01-05

**Authors:** Annina Lyly, Anu Laulajainen‐Hongisto, Heikki Turpeinen, Seija I. Vento, Jyri Myller, Jura Numminen, Saara Sillanpää, Johanna Sahlman, Paula Kauppi, Sanna Toppila‐Salmi

**Affiliations:** ^1^ Inflammation Center, Skin and Allergy Hospital University of Helsinki and Helsinki University Hospital Helsinki Finland; ^2^ Department of Otorhinolaryngology—Head and Neck Surgery University of Helsinki and Helsinki University Hospital Helsinki Finland; ^3^ Department of Otorhinolaryngology Päijät‐Häme Central Hospital Lahti Finland; ^4^ Department of Otorhinolaryngology University of Tampere, Faculty of Medicine and Life Sciences and Tampere University Hospital Tampere Finland; ^5^ Department of Otorhinolaryngology Kuopio University Hospital Kuopio Finland; ^6^ Medicum, Haartman Institute University of Helsinki Helsinki Finland

**Keywords:** asthma, CRS, disease control, eosinophilia, nasal polyps, N‐ERD

## Abstract

**Background:**

Nonsteroidal anti‐inflammatory drug (NSAID) exacerbated respiratory disease (N‐ERD) is a triad with asthma, chronic rhinosinusitis with nasal polyps, and NSAID intolerance. Uncontrolled N‐ERD forms a major public health problem due to frequent and difficult‐to‐treat exacerbations and/or requiring putatively frequent endoscopic sinus surgeries (ESS). Our aim was to study factors affecting control of N‐ERD.

**Methods:**

Retrospective patient record data (patient characteristics, prior sinus surgeries, follow‐up data in 2020) from 167 N‐ERD patients undergoing consultation at three tertiary hospitals from 2001 to 2017 was used. Outcome measurements reflecting uncontrolled N‐ERD were revision ESS, corticosteroids/biological therapy, and antibiotic courses during 2016–2020. Associations were analyzed by using nonparametric tests, Cox's proportional hazard, and binary logistic regression models.

**Results:**

Nasal polyp eosinophilia increased the risk of revision surgery during the follow‐up (adjusted hazard ratio [aHR] 3.21, confidence interval 1.23–8.38). Also baseline oral corticosteroids (OCS; HR, 1.73, 1.04–2.89) and baseline surgery without total ethmoidectomy increased the risk of revision ESS (HR, 2.17, 1.07–4.42) in unadjusted models. In addition, both baseline OCS (adjusted odds ratio [aOR] 2.78, 1.23–6.26) and a history of ≥4 previous ESS (aOR, 2.15, 0.98–4.70) were associated with the use of OCS/biological therapy during the follow‐up, but not with high number of antibiotics.

**Conclusions:**

Nasal polyp eosinophilia, baseline OCS, and a history of recurrent ESS predict uncontrolled N‐ERD. These factors might be clinically useful in risk‐estimation of uncontrolled disease and for organizing follow‐ups. Prospective cohort studies with larger sample size are needed to further study the factors affecting the upper airway control of N‐ERD.

## Graphical abstract

The patient record data of 167 patients with NSAID‐exacerbated respiratory disease was analyzed to study factors affecting the disease control. Nasal polyp eosinophilia increased the risk of revision surgery, and baseline oral corticosteroids and a history of four or more endoscopic sinus surgeries were associated with poor disease control during the follow‐up.

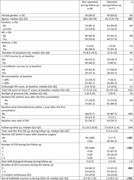

**Abbreviations**
AD: aspirin desensitization
AR: allergic rhinitis
ATAD: aspirin treatment after desensitization
CRS: chronic rhinosinusitis
CRSwNP: chronic rhinosinusitis with nasal polyps
CT: computed tomography
ENT: ear nose and throat
ESS: endoscopic sinus surgery(ies)
LM: Lund‐Mackay
N‐ERD: NSAID‐exacerbated respiratory disease
OCS: oral corticosteroid(s)

## 1 INTRODUCTION

Nonsteroidal anti‐inflammatory drug (NSAID) exacerbated respiratory disease (N‐ERD) is an inflammatory disease of the airways usually including a triad of chronic rhinosinusitis with nasal polyposis (CRSwNP), asthma, and hypersensitivity to acetylsalicylic acid (ASA) and other NSAIDs.^1^, ^2^ Patients with N‐ERD have severe eosinophilic hyperplastic inflammation, tissue remodeling, and fibrosis of both the paranasal sinuses and lower airways, and abnormalities of the cyclo‐oxygenase (COX) pathway.^1^, ^3^, ^4^ N‐ERD is slightly more common in females, it typically starts around the age of 30, and has an estimated prevalence of 9% in patients with asthma.^5^, ^6^

Chronic rhinosinusitis (CRS) is one of the most common chronic adult health problems and causes severe impact on the quality of life.^7^, ^8^, ^9^ CRS is defined as chronic inflammation (≥12 weeks) of the nose and paranasal sinuses characterized by rhinitis symptoms and it is diagnosed with nasal endoscopy and computed tomography (CT).^9^, ^10^, ^11^, ^12^, ^13^ CRS phenotypes include CRSwNP and CRS without nasal polyps (CRSsNP). In the Western world, 6–11% of the general population has CRS, and 4% has CRSwNP; 8–26% of CRSwNP patients have N‐ERD.^7^, ^8^, ^14^, ^15^, ^16^

CRSwNP treatment includes saline irrigations, nasal steroids, oral steroids, oral antimicrobials, and surgery if conservative treatment is not sufficient.^2^, ^14^ Especially in N‐ERD patients, CRSwNP may lead to recurrent, multiple sinus surgeries.^17^, ^18^ A study in a tertiary referral center showed that N‐ERD patients had undergone two‐fold more sinus surgeries (*p* < .001) and were significantly younger at the time of their first surgery than were CRSwNP patients without N‐ERD.^19^

Moderate‐to‐severe asthma is common in N‐ERD patients. Severe asthma, with the need for high dose inhaled corticosteroid treatment, steroid bursts and/or continuous OCS, and increased risk for healthcare visits, hospitalizations, and intubations due to asthma exacerbations are more common in N‐ERD patients than other asthmatics.^2^, ^3^, ^5^ An escalation in CRS symptoms and management (acute symptoms of paranasal sinuses, and systemic use of antibiotics or corticosteroids) is considered a CRS exacerbation,^14^, ^20^, ^21^, ^22^ and it often associates with poor asthma control.^23^

Aspirin treatment after desensitization (ATAD) can be considered if an N‐ERD patient has insufficient response to other CRS treatments, has insufficient control of asthma symptoms despite the use of asthma medications, or needs ASA or NSAID treatment for other medical reasons.^2^, ^24^ ATAD has a beneficial effect on disease control in N‐ERD patients. A retrospective ATAD study showed improvements in symptoms, quality of life, need for rescue therapy for airway symptoms, and improvement in the sense of smell or taste.^25^ Another report showed beneficial effects of ATAD on the disease control of both rhinosinusitis and asthma.^26^

In the past few years, biologic therapy modulating the type 2 inflammation present in eosinophilic asthma, but also in CRSwNP, has become intriguing for rhinologists also. At the moment, anti‐IL4/IL13 and anti‐IL5 have been shown to be effective and are the recommended treatment options in otherwise suitable CRSwNP patients.^9^

We and other study groups have previously shown that revision ESS is associated with gender, young adults, smoking, allergic rhinitis, previous ESS, occupational exposure, presence of nasal polyps, need for systemic medication, asthma, and N‐ERD.^18^, ^27^, ^28^, ^29^, ^30^, ^31^, ^32^, ^33^, ^34^ In our unpublished data, we have recognized that N‐ERD is a potential predictor of uncontrolled CRSwNP compared to controlled CRSwNP.

N‐ERD forms a long‐lasting health problem due to frequent and difficult‐to‐treat exacerbations and frequent surgeries.^35^ Our recent retrospective follow‐up study showed ATAD's weak effect on N‐ERD control.^36^ The aim of the current study was to evaluate factors that predict disease control among CRSwNP + N‐ERD patients.

## 2 METHODS

### 2.1 Subjects

This retrospective follow‐up study was carried out in the Skin and Allergy Hospital and Department of Otorhinolaryngology at Helsinki University Hospital, as well as Departments of Otorhinolaryngology at Tampere and Kuopio University Hospitals, between 2001 and 2020. The study (No. 31/13/03/00/2015) was approved by the ethical committee of Hospital Districts.

This study involved 167 N‐ERD patients undergoing ENT consultation between 2001 and 2017. Inclusion criteria were N‐ERD (e.g. a patient‐record documented history of wheeze/cough/naso‐ocular symptoms after ingestion of NSAID ± positive ASA challenge test result), follow‐up data available, and patient record data of nasal endoscopic signs of NPs at the baseline visit or baseline surgery. CRSwNP was diagnosed according to the European Position Paper on CRS and NPs.^14^ Exclusion criteria were age under 16 years and unavailable or incomplete data of ESS at baseline and/or during the follow‐up.

Asthma was diagnosed according to Global Initiative for Asthma, for example, patient record documentation was based on typical history and asthma symptoms, and findings of lung function test of at least 15% improvement with bronchodilator test in spirometry (in forced expiratory flow volume in one second FEV1 or in forced vital capacity FVC) and/or recurrent 20% diurnal variation in PEF monitoring or recurrent 15% bronchodilator response in PEF monitoring or positive methacholine challenge test (moderate‐to‐severe bronchial hyperresponsiveness). N‐ERD diagnosis was based on a positive history of wheeze/cough or naso‐ocular symptoms after intake of NSAID.^37^ N‐ERD diagnosis was additionally based on a positive reaction (wheeze and/or naso‐ocular reaction) after intake of ASA at the hospital among the 96 subjects who underwent ATAD at baseline.^36^

The background data (age, gender, smoking habits, allergic rhinitis, asthma, baseline use of OCS/antibiotic courses, baseline start with ATAD, duration of symptoms, previous ESS, baseline endoscopic NP score, baseline LM score of sinus CT scans) were collected from the patient records. We also collected data of baseline ESS (defined as surgery performed within 12 months from the sinus CT scans taken during the baseline visit of sinus surgical consultation); and previous surgery (defined as ESS performed before the baseline visit of sinus surgical consultation).

The follow‐up data were collected in 2018–2020; mean (min–max) 7.5 (0.9–16.2) years after the consultation visit. The collected follow‐up data included revision ESS during the follow‐up; information regarding start with biological therapy during the follow‐up, continuous OCS during the follow‐up, and purchased, doctor‐prescribed OCS and antibiotic courses (due to exacerbation of CRSwNP and/or asthma) during 2016–2020, was obtained from the national electronic prescription system. Uncontrolled N‐ERD during the follow‐up was in interest. The signs of uncontrolled N‐ERD during the follow‐up were determined by using three outcome measurements: (1) revision ESS; (2) more than one purchased OCS courses/year and/or continuous OCS and/or biological therapy; (3) more than two purchased oral antibiotic courses/year.

### 2.2 Statistical analyses

Statistical analysis was carried out by the SPSS Base 15.0 Statistical Software Package (SPSS Inc.). Associations between follow‐up surgery rate and background/follow‐up factors were assessed with Fisher's exact test (dichotomous), Kruskal–Wallis, and Mann–Whitney *U* tests (continuous). Cox's proportional hazards models were used to evaluate the hazard ratio (HR) of the revision surgery rate between different background variables. Each predisposing factor was modeled separately. The background variables that were associated with purchased of corticosteroids/biological therapy or antibiotics during follow‐up were separately modeled by binary logistic regression. The background variables that were associated significantly with revision surgery or use of corticosteroid/biological therapy/antibiotics, were entered into the multivariable model. *p* values of <.05 were considered statistically significant.

## 3 RESULTS

The N‐ERD patients who underwent ESS during the follow‐up were statistically significantly younger and purchased more OCS and/or antibiotics during the follow‐up (Table 1). Other baseline factors did not differ between operated and nonoperated during follow‐up groups (Table 1).

**Table 1:** Patient background and follow‐up characteristics

	Nonoperated during follow‐up	Operated during follow‐up	
	*n *= 90	*n* = 77	*p*
Gender, female, *n* (%)	54 (60.0)	49 (63.6)	.64
Age, median (Q1–Q3) (year)	48.6 (39–59)	45.3 (35–53)	.027
Smokers, *n* (%)			
No	74 (85.1)	62 (83.8)	.83
Current	13 (14.9)	12 (16.2)	
AR, *n* (%)			
No	40 (44.4)	32 (42.1)	.88
Yes	50 (55.6)	44 (57.9)	
Asthma, *n* (%)			
No	3 (3.4)	2 (2.6)	1.0
Yes	86 (96.6)	75 (97.4)	
Duration of symptoms (years), median (Q1‐Q3)	15 (8.5–24.5)	12 (5–23)	.40
≥ 1 OCS course/years, at baseline, *n* (%)			
No	46 (53.5)	29 (39.7)	.11
Yes	40 (46.5)	44 (60.3)	
≥4 Antibiotic courses/years, at baseline, *n* (%)			
No	49 (62.8)	37 (56.9)	.50
Yes	29 (37.2)	28 (43.1)	
NP eosinophilia, at baseline, *n* (%)			
<30%	12 (34.3)	7 (16.3)	.11
≥30%	23 (65.7)	36 (83.7)	
Endoscopic NP score, at baseline, median (Q1–Q3)	5 (3.75‐6)	5.5 (4–6)	.11
Total LM score of sinus CT scans, at baseline, median (Q1–Q3)	17.5 (14–21)	20 (15–23)	.056
Number of previous ESS, median (Q1–Q3)	2 (0‐3.25)	2 (0–4)	.76
Baseline ESS within 1 year after the first consultation, *n* (%)			
No	17 (18.9)	12 (15.6)	.68
Yes	73 (81.1)	65 (84.4)	
Baseline total ethmoidectomy within 1 year after the first consultation, *n* (%)			
No	66 (76.7)	64 (87.7)	.099
Yes	20 (23.3)	9 (12.3)	
Baseline start with ATAD, *n* (%)	51 (56.7)	43 (59.7)	.75
Follow‐up time (years), median (Q1–Q3)	6.1 (4.2–10.6)	7.9 (4.9–11.4)	**.049**
Time until the first ESS (years), during follow‐up, median (Q1–Q3)^a^	‐	3 (1.5–4.5)	‐
Revision ESS within 5 years after baseline surgery^b^			
No	59 (100)	5 (7.7)	**< .001**
Yes	0 (0)	60 (92.3)	
Number of ESS during the follow‐up			
0	90 (100)	0 (0)	**< .001**
1	0 (0)	52 (67.5)	
2	0 (0)	20 (26.0)	
≥3	0 (0)	5 (6.5)	
Start with biological therapy during follow‐up	3 (3.3)	2 (2.6)	1.0
Number of OCS courses/years during the follow‐up, *n* (%)			
0	34 (44.2)	13 (21.0)	**.002**
≥1–2	20 (26.0)	33 (53.2)	
3 and/or continuous OCS	23 (29.9)	16 (25.8)	
No. of antibiotic courses/years during 2016–2019, median (Q1–Q3)	0.5 (0–1.25)	1.0 (0.5–2)	**< .001**

*Note: p* values by Fisher's exact test (dichotomous variables) or Kruskal–Wallis and Mann–Whitney *U* test (continuous variables). Q1 = 25% percentile, Q3 = 75% percentile. Bold values denote statistical significance at the *p* < .05 level.

Abrreviations: AD, aspirin desensitization; AR, allergic rhinitis; ASA, aspirin; ATAD, ASA treatment after desensitization; CRS, chronic rhinosinusitis; N‐ERD, patient‐reported NSAID‐exacerbated respiratory disease; NP, nasal polyps; OCS, oral corticosteroid.

^a^ All patients were included.

^b^ Only the group which underwent baseline ESS within 1 year after the baseline consultation.

The proportion of the N‐ERD patients who underwent baseline ESS was 138 of 167 (82.6%). This patient group data were analyzed in univariate and multivariable Cox's proportional hazard models to analyze the background variables fitted for the need for revision ESS during the follow‐up. Nasal polyp eosinophilia (≥30% of all leukocytes) increased the risk of revision ESS during the follow‐up (HR, 2.67; CI, 1.11–6.48) in univariate models (Table 2). Also baseline OCS (HR, 1.73; 1.04–2.89), and baseline surgery without total ethmoidectomy increased the risk of revision ESS (HR, 2.17; 1.07–4.42), in the univariate model (Table 2). When entering these three baseline variables into the multivariable model, only NP eosinophilia was significantly associated with the risk of revision ESS during the follow‐up (aHR, 3.21; 1.23–8.38; Table 2).

**Table 2:** Univariate and multivariable Cox's proportional hazard models for the background variables analyzed fitted for the need for revision ESS during the follow up of mean (min–max) 7.2 (0.9–16.2) years

	*N* All	*N* (%) ESS	HR (95% CI)	Adjusted HR (95% CI)
Gender				
Female	86	39 (45.3)	1	
Male	52	26 (50.0)	1.10 (0.67–1.80)	
Age				
≥47 years	62	27 (43.5)	1	
<47 years	76	38 (50.0)	1.12 (0.68‐1.83)	
LM score				
<16/24	43	17 (39.5)	1	
≥16/24	83	41 (49.4)	1.33 (0.75–2.34)	
NP score				
<4/8	21	6 (28.6)	1	
≥4/8	97	49 (50.5)	2.00 (0.85–4.67)	
NP eosinophilia				
<30%	17	6 (35.3)	1	1
≥30%	47	31 (66.0)	**2.67 (1.11–6.48)**	**3.21 (1.23‐8.38)**
Baseline OCS courses/year				
<1	66	24 (36.4)	1	1
≥1	65	38 (58.5)	**1.73 (1.04‐2.89)**	1.03 (0.53–2.01)
Baseline ab courses/year				
<4	75	35 (46.7)	1	
≥4	42	20 (47.6)	1.09 (0.63‐1.90)	
Baseline total ethmoidectomy				
Yes	102	9 (32.1)	1	1
No	28	52 (51.0)	**2.17 (1.07–4.42)**	2.50 (0.75–8.35)
Baseline start with ATAD				
Yes	75	35 (46.7)	1	
No	63	30 (47.6)	0.92 (0.56–1.51)	

*Note:* Only the N‐ERD subjects who underwent ESS at the baseline (*n* = 138) were included. Bold values denote statistical significance at the *p* <* *.05 level. The second model is a multivariable model adjusted by the background variables that were associated with revision ESS at *p* < .05 level in the first model.

Abbreviations: ab, antibiotic; ATAD, AD followed by ASA treatment after desensitization; CI, confidence interval; ESS, endoscopic sinus surgeries; HR, hazard ratio; NERD, nonsteroidal anti‐inflammatory drug exacerbated respiratory disease; OCS, oral corticosteroid.

The proportion of the N‐ERD patients of whom we had data of medication during follow‐up was 140 of 168 (86.4%). This patient group data were analyzed in univariate and multivariable logistic regression models to analyze the background variables that were associated with OCS/biological therapy during follow‐up. Both baseline OCS (OR 2.87, 1.29–6.41) and a history of ≥4 previous ESS (OR, 2.19; 1.04–4.63) were associated with the use of OCS/biological therapy during the follow‐up in univariate models (Table 3). When entering these two baseline variables into the multivariable model, both were significantly associated with the use of OCS/biological therapy during the follow‐up (aOR, 2.78, 1.23–6.26; aOR, 2.15, 0.98–4.70, respectively; Table 3). In addition, the variables of this patient group were identically analyzed in univariate logistic regression models by using “>2 antibiotic courses/year during follow‐up” as an outcome measurement. None of the background factors were significantly associated with purchased antibiotic courses in these models (*p* > .05, data not shown).

**Table 3:** Univariate and multivariable binary logistic regression models for the background variables analyzed fitted for the number of purchased doctor‐prescribed OCS

	*N* all	*N* (%) outcome	OR (95% CI)	Adjusted OR (95% CI)
Gender				
Female	88	25 (28.4)	1	
Male	52	16 (30.8)	1.12 (0.53–2.37)	
Age				
≥47 years	70	16 (22.9)	1	
<47 years	70	25 (35.7)	1.88 (0.89–3.94)	
Previous ESS				
<3	90	21 (23.3)	1	1
≥3	50	20 (40.0)	**2.19 (1.04–4.63)**	**2.15 (0.98–4.70)**
LM score				
<16/24	49	11 (22.4)	1	
≥16/24	82	29 (35.4)	1.89 (0.84–4.25)	
NP score				
<4/8	21	6 (28.6)	1	
≥4/8	104	32 (30.8)	1.11 (0.40–3.13)	
NP eosinophilia				
<30%	17	3 (17.6)	1	
≥30%	54	16 (29.6)	1.97 (0.50–7.79)	
Baseline OCS courses/year				
<1	60	11 (18.34)	1	1
≥1	74	29 (39.2)	**2.87 (1.29–6.41)**	**2.78 (1.23–6.26)**
Baseline ab courses/year				
<4	76	18 (23.7)	1	
≥4	50	20 (40.0)	2.15 (0.99–4.66)	
Baseline total ethmoidectomy				
Yes	22	7 (31.8)	1	
No	118	34 (28.8)	0.87 (0.33–2.32)	
Baseline start with ATAD				
Yes	80	27 (33.8)	1	
No	60	14 (23.3)	0.60 (0.28–1.27)	

*Note:* The search for prescription data was performed from nation‐wide electronic prescription database during 2016–2020. The outcome measurement was >1 OCS courses per year or continuous OCS or biological therapy started during the follow‐up. Only the N‐ERD subjects with available e‐prescription data (*n* = 140) were included. Bold values denote statistical significance at the *p* < .05 level. The second model is a multivariable model adjusted by the background variables that were associated with follow‐up corticosteroid or antibiotic use at *p* < .05 level in the first model.

Abbreviations: ab, antibiotic; ATAD, ASA treatment after desensitization; CI, confidence interval; NERD, nonsteroidal anti‐inflammatory drug exacerbated respiratory disease; OCS, oral corticosteroid; OR, odds ratio.

## 4 DISCUSSION

We analyzed the background characteristics and follow‐up data of N‐ERD patients focusing on their upper airway control. Outcome measurements involved patient record data of revision ESS, use of antibiotics, and use of OCS/biologicals in the follow‐up. It was known from previous studies with follow‐up times up to 20 years, that CRSwNP patients with N‐ERD have high recurrence rate and are more likely to be operated several times.^35^, ^38^ In our cohort, approximately half of the patients underwent revision surgery during the follow‐up time (mean 7.5 years). We wanted to reveal possible predicting factors for poor disease control in this already selected subgroup of CRSwNP patients and found that tissue eosinophilia (30% or more) correlated with worse disease control and increased risk of revision surgery.

Tissue eosinophilia has earlier been reported to predict poorer outcome after ESS.^39^ A report concerning 77 eosinophilic CRSwNP patients from Japan showed that poor disease control in 1‐year follow‐up was associated with younger age and higher tissue eosinophilia, together with higher LM score.^40^ Chinese colleagues analyzed nearly 400 CRSwNP patients, and reported a cut‐off value of 27% of tissue eosinophilia and a count of 55 eosinophils per high power field as predicting factors for polyp recurrence within 2 years after surgery.^41^ Our result is in line with this finding, even though our cohort was even more selected with patients most likely presenting eosinophilic inflammation.

In previous retrospective reports concerning nonselected CRS‐patients, factors predicting revision surgery included previous surgeries, the existence of nasal polyps, and the use of corticosteroids among other factors such as female gender.^18^, ^33^ In a study concerning patients with nasal polyps, the factors predicting earlier revision surgery were smoking and operating technique (preserving the middle turbinate).^31^ We could not find a correlation with smoking or gender in our results. In the study by Wu et al.,^31^ the tissue eosinophilia was reported as either existing or none existing, and it did not show a correlation with the time of revision surgery. Another study investigating the prevalence of polyp recurrence after surgery identified previous surgery and high polyp grade as risk factors for more rapid recurrence.^17^ We did not see the baseline polyp score or LM score affecting the need for revision surgery. The polyp score was relatively high in our cohort at the baseline already, 5 and 5.5, which might explain this result.

Comparison between different operating techniques is difficult keeping in mind the vast range of techniques from simple polypectomy to Draf III procedures. Alsharif et al.^42^ compared different ESS techniques in a retrospective case‐control study on patients with type 2 CRSwNP patients, and found out that removing the mucosa from all paranasal sinuses resulted in better disease control than the traditional mucosa‐sparing technique. They speculated that removing the inflammatory tissue completely would impact the natural course of the disease. In our data, the baseline total ethmoidectomy was related to better disease control in univariate model, but the result did not replicate in multivariate model. Prospective, randomized studies about baseline surgery techniques are needed to clarify their role in the disease control in this subgroup of CRSwNP patients.

In N‐ERD patients with difficult symptoms, ATAD can be considered. Our retrospective follow‐up study on ATAD's efficacy among these subjects has shown that the ATAD's discontinuation rate was high, over 60%, and that ATAD was not associated with use of OCS or with revision ESS during the follow‐up.^36^ The scope of the current study was mainly on other factors behind controlled or uncontrolled NERD, nevertheless, we detected again that ATAD was not associated with revision ESS or use of OCS/biologics during the follow‐up. Hence, it could be speculated that factors related to poor disease control at baseline (such as a history of eosinophilia, OCS, or recurrent ESS) might impact more on disease control during follow up than start with ATAD. However, prospective controlled follow‐up studies are needed to better evaluate this.

The use of biologicals was scarce in our cohort (5/167 patients) and there was no difference between the groups. Concerning the time of the data collection, the indication for initiating biological treatment was most likely asthma, as CRSwNP got the official indication for the first biological only in late 2019. In the future, patients with N‐ERD are likely to be among the first when considering the initiation of biologicals for CRSwNP patients.

When assessing the factors affecting the need for OCS courses during the follow‐up, we found that the amount of baseline OCS as low as one per year predicted corticosteroid use also for the future. This might be linked to higher eosinophilic inflammation with those who have benefitted from OCS—it suppresses the inflammation very effectively and the patients get relief quickly, making the medicine an attractive choice. On the contrary, the amount of previous surgeries predicting OCS use was quite high, four, or more. This also indicates for an active inflammatory disease—if the patient has already been operated several times and the symptoms persist or return quickly after operations, the use of OCS courses is needed to keep the symptoms bearable. This, together with the finding of high eosinophilia in the tissue as a predicting marker for poor disease control, speaks for the need of systematic evaluation of overall type 2 inflammation activity of the patients initially and keeping those patients with high grade of inflammation in closer control.

There are some limitations worth discussing in our study. Due to its retrospective nature, the diagnostics or treatments of N‐ERD were not standardized, making the comparison with other studies more challenging. Ninety percent of the population had a history of using baseline intranasal corticosteroids, 4% were not using, and 6% were lacking the data in patient records. We acknowledge that data were lacking concerning treatment adherence and/or reason for not using intranasal corticosteroids. There might have been limitations in the variables collected from the patient records and the lack of quality‐of‐life measurements limits the analyses. Some patients with exacerbations may also have been treated elsewhere. However, public medical care covers over 90% of operations in Finland.^43^ In addition, those finishing hospital follow‐ups were followed by electronic prescriptions of OCS/antibiotics/biologic, which would decrease misclassification bias. All in all, our study provides information on factors behind uncontrolled N‐ERD in real‐life setting.

## 5 CONCLUSION

Nasal polyp eosinophilia, baseline corticosteroid course(s), and a history of recurrent ESS predict uncontrolled N‐ERD. Prospective cohort studies with larger populations are needed to evaluate the risk factors of uncontrolled N‐ERD.

## CONFLICT OF INTERESTS

Sanna Toppila‐Salmi reports a grant of GSK and consultancies for ERT, Novartis, Sanofi Pharma, and Roche Products outside the submitted work. Anu Laulajainen‐Hongisto reports a grant from the Orion Research Foundation, outside of the submitted work. The other authors declare that there are no conflict of interests.

## AUTHOR CONTRIBUTIONS

Annina Lyly, Anu Laulajainen‐Hongisto, and Sanna Toppila‐Salmi wrote the article. Sanna Toppila‐Salmi planned the study and collected and analyzed the data. Anu Laulajainen‐Hongisto, Heikki Turpeinen, Seija I. Vento, Jura Numminen, Saara Sillanpää, Jyri Myller, Johanna Sahlman participated in data collection. All the authors critically reviewed the final article text.
